# Hospitals, Their Location, Etc.

**Published:** 1880-04

**Authors:** 


HOSPITALS, THEIR LOCATIONS, ETC.—We hoped, until a day or two ago, to have written at some length upon the above subject in this issue of our journal, but find that lack of space will restrict us now to a brief notice only. However, the subject is a large one, and perhaps our readers will be better pleased to receive it in instalments than to have it all at once. As our remarks should very naturally include hospitals in and near Baltimore, we purpose to devote a few words to one or two of them in this number of the journal. First: the location of several hospitals appertaining to, and near the city, is all that could be expected or desired; their construction, elevation, freedom from noise and confusion, and general hygienic surroundings being admirably suited and adapted to the comfort and convenience of the sick and suffering who find asylum and treatment within their walls. But some of them require certain changes of management or discipline in order to fit them properly for the full work of general hospitals, and consequently for all classes and conditions of persons. Bayview Asylum, for example, is a good illustration of what we have been saying, as it contains all the external requisites named, and even more, for the landscape and water views are unusually fine, probably not surpassed, taken altogether, by a hospital anywhere; but unfortunately it is controlled, in the selection of its trustees and other officers, by political and party influence, and all who are acquainted with the working of such machinery by a dominant party understand well what to expect in such a state of things; and until this power can be lessened or modified in an appreciable degree, the hospital will not become every thing it is susceptible of being made. The alliance of politics and science is not a proper or congenial one, and until they are divorced there will not be every blessing bestowed upon the sick that a non-partisan management would be likely to confer. But as a more opportune occasion may occur for discussing the subject in the future, we will proceed to say a few words of St. Vincent's Hospital ere we conclude our remarks in this issue.

This hospital, like Bayview, is admirably located, and surrounded by accessories which should go far toward rendering it a suitable place for such an institution, save in one important particular. It is owned and under the care and control of the Roman Catholic communion, and although receiving patients from all or no religious denominations—if we are correctly informed—its internal affairs are managed and controlled and the nursing done by the Sisters of Charity of that church. Were it to restrict its admission of patients to those of that religious faith only, we know of no reasonable objection that could be urged or adduced to its prejudice or disadvantage. But while we recollect that this is and has been a Protestant country, and mainly under Protestant rulers from the first establishment of the government, and though it does not attempt to interfere with any religious creed or denomination in the mode or manner of worship it might choose to adopt or practise, yet it must appear evident to an ordinarily intelligent mind that a sick man brought up in strict conformity to the requirements of the Protestant faith, for example (as the large majority of our people are), would very naturally prefer the ministrations of Protestant nurses, physicians, and clergy, to the Sisters of Charity, however kind they might be; or of priests, though devout and attentive to the duties of their office, as these would very naturally remind him in their insignia and drapery, if in no other way, that they are members of a communion in which he can have at best but little faith. The mind of a sick man is not in a condition to be disturbed or interfered with by religious opinions opposed to those in which he was born and reared; and even the insignia of a creed that antagonizes—and the remark applies to any creed—the faith in which he was nurtured is not calculated to produce a pleasing or soothing effect upon his mind, or upon the disease under which he might be suffering. We believe in the great Atonement, and try to be catholic in our relations and conduct toward our fellow-men; but simple justice to the sick who find their way into such institutions requires us to animadvert as we have done in connection with this matter of commingling the various creeds in the persons of the sick under the government and control of one particular denomination. Political parties and denominational distinctions are probably both necessary in a democratic republican country like ours, and should not, therefore, be discountenanced or discouraged among the well, as it is likely they conspire to the healthy growth of state and church, when conducted with proper spirit and decorum of conduct. But they should in nowise be permitted to enter, even in the most modified form, the sacred precincts of institutions dedicated to the care of the sick and the dying. But for the present, *verbum sat*, etc.

